# The Role of Physical Activity in the Reduction of Generalised Anxiety Disorder in Young Adults in the Context of COVID-19 Pandemic

**DOI:** 10.3390/ijerph191711086

**Published:** 2022-09-05

**Authors:** Ewelina Czenczek-Lewandowska, Justyna Leszczak, Justyna Wyszyńska, Joanna Baran, Aneta Weres, Bogumił Lewandowski

**Affiliations:** 1 Institute of Health Sciences, Medical College, University of Rzeszów, ul. Kopisto 2a, 35-959 Rzeszow, Poland; 2 Institute of Medical Sciences, Medical College, University of Rzeszów, ul. Kopisto 2a, 35-959 Rzeszow, Poland

**Keywords:** physical activity, young adults, generalised anxiety, COVID-19

## Abstract

Physical activity is critically important not only for physical but also for mental health. Exercise may be a beneficial form of therapy for young adults with anxiety disorders. The global outbreak of the COVID-19 pandemic adversely affected the public, including young adults, in terms of their mental well-being and opportunities for physical activity. The study aimed to identify the influence of physical activity (PA) on generalised anxiety in young adults. It also assessed the changes which occurred in the level of PA and in generalised anxiety in young adults as a result of COVID-19 pandemic. A cross-sectional survey was carried out online with 506 young adults aged 18 to 34 years (=24.67 years ± 4.23 years). Respondents provided two answers to each question, i.e., information relating to the last 7 days during the pandemic (first hard lockdown), and to a period of 7 days before the pandemic (retrospective). The levels of physical activity were measured using 7-item International Physical Activity Questionnaire-Short Form (IPAQ–SF), whereas the level of generalised anxiety was assessed using the Generalised Anxiety Disorder 7 (GAD-7) questionnaire. During the pandemic there was a significant correlation between the level of generalised anxiety and the level of physical activity reported by the respondents (*p =* 0.048). A higher level of physical activity corresponded to lower level of generalized anxiety in young adults. During the pandemic, young adults spent significantly less time performing physical activity (8752.5 vs. 6174.6 metabolic equivalents (MET) min/week, *p <* 0.001), they spent more time engaging in sedentary behaviours (Me = 240 vs. Me = 300 min/day, *p <* 0.001), and they walked much less (from Me = 6930.0 (MET) min/week vs. 3861.0 (MET) min/week (44.29% decrease). Furthermore, after the outbreak of the pandemic the level of perceived generalised anxiety increased significantly (*p <* 0.001). Physical activity may play an important role in reducing anxiety disorder in young adults. During the COVID-19 pandemic young adults were significantly less involved in PA, which adversely affected their physical and mental condition. The importance of sufficient PA should be emphasised during this specific period, particularly among young adults.

## 1. Introduction

Regular physical activity (PA) is one of the most effective non-pharmacological treatments of noncommunicable diseases (NCDs), including mental disorders, such as depression and anxiety. Both are increasingly common especially among younger populations [1]. Various biochemical, physiological, and psychological hypotheses have been proposed to explain the beneficial effects of PA on mental health disorders. One of them refers to the protective role of PA in dysregulations in the hypothalamic–pituitary–adrenal axis (HPA), the autonomic nervous system, and immune system, which play an important role in mediating the stress response. Another biological mechanism is related to excessive and systemic inflammation as a result of chronic psychological stress. It is established that PA has a strong anti-inflammatory effect as well as a positive influence on behavioural and metabolic resilience. In addition, regular physical exercise improves the neuroplasticity and stimulates the production of neurotrophic factors, especially brain-derived neurotrophic factor (BDNF), endorphins, monoamines (serotonin and epinephrine), and phenylethylamine. All of these processes undoubtedly affect improvement of mood, motivation, self-esteem, memory function, and slowing down of cognitive decline, and in consequence, delaying an onset of mental problems [2,3,4].

Individuals with severe mental disorders and depression are 50% less likely to meet worldwide physical activity guidelines, they spend nearly 8 h per day in a sedentary position and, on average they spend 38 min less doing moderate physical activity compared to their peers [5,6]. It was reported that people with depression or anxiety disorders are more likely to create barriers that prevent them from engaging in sufficient physical activity. These may include internal lack of motivation resulting from poor mood, a sense of fatigue, lack of willingness to meet with peers, and poor awareness of the benefits from PA for health [7]. Additional hazards include unfavourable environment-related factors, such as difficult access to places for recreation. The outbreak of the global COVID-19 pandemic severely limited opportunities for PA. The first hard lockdown was particularly difficult, because in addition to restrictions on movement, it was associated with significant fear experienced by the public with regard to this unfamiliar threat to health and life [8]. The global pandemic not only led to increased risk of death due to coronavirus infection but also exacerbated many previously existing public health issues, such as generalised anxiety disorder (GAD), affecting young populations as well. The data reported so far reflects relatively high rates of symptoms of anxiety induced by the pandemic, ranging from 6.33% to 50.9% depending on the location, the populations below 40 years of age being particularly at risk [9]. It is estimated that 75% of all mental disorders start to develop at an age of 24 years [10]. Young adulthood is a major transition period, associated with numerous changes in one’s life, such as increasing financial and emotional independence. This period may be even more challenging for individuals experiencing anxiety disorder [11]. Some researchers have observed that young adults are frequently overlooked in the context of anxiety disorder, because as a rule it is believed they have full physical and mental capacities. Anxiety disorders in young adults are frequently undiagnosed and untreated, due to the lack of screening procedures in this age group. Mental health treatment is most frequently provided to children or elderly individuals [12]. In recent years, international guidelines related to the standards of mental disorder treatment have started to take into account PA, as an integral part of complex healthcare provided to patients with mental disorders, hence it is worthwhile to investigate the role of PA in the reduction of anxiety disorders [13].

The study aimed to identify the influence of physical activity (PA) on generalised anxiety in young adults. It also assessed the changes which occurred in the level of PA and the experience of generalised anxiety in young adults, in the period before the outbreak of COVID-19 pandemic compared to the time during the first hard lockdown at the start of the pandemic.

## 2. Materials and Methods

### 2.1. Study Design

The cross-sectional study was carried out in south-eastern Poland in the initial period of the global COVID-19 pandemic, in 2020, between weeks 2 and 4 of the hard lockdown, imposing significant restrictions on movement. A survey was conducted using an online questionnaire, since this was the only method enabling contact with respondents, due to the restrictions. The survey was based on standardised questionnaires shown to have good reliability and construction validity. Respondents provided two answers to each question, with reference to the period of 7 days before the outbreak of the pandemic (retrospective assessment) and the period of the first hard lockdown; as a result it was possible to assess physical activity and generalised anxiety in those two periods of time, and ultimately to perform the related comparative analyses. Eligibility criteria included age between 18 and 34 years, place of residence in south-eastern Poland, informed consent to participate in the study (given before the start of the survey), fully completed and returned questionnaire, and lack of reported and diagnosed chronic diseases, including mental problems. The study was approved by the Ethics Committee of the University of Rzeszow (ref. no. 2/02/2019).

### 2.2. Study Sample

The analyses took into account 506 young adults aged 18–34 (=24.67 years ± 4.23 years). Based on the related data showing that the population of south-eastern Poland comprises around 250,000 adults in this age group, and assuming a confidence level of 95%, and a 5% margin of error, it was calculated that the required sample should include at least 384 individuals. The number of respondents taken into account in the analyses exceeded this minimum sample size.

### 2.3. Measures

#### 2.3.1. Physical Activity and Sedentary Behaviours (IPAQ–SF)

The level of physical activity in these two periods was determined based on responses given to questions in the 7-item International Physical Activity Questionnaire-Short Form (IPAQ–SF), which is the most widely used tool enabling assessment of physical activity [14]. The respondents answered 7 questions covering all kinds of physical activity associated with daily life, work and leisure. They subjectively assessed average daily time spent sitting, walking, and engaging in moderate and vigorous physical activity. They were only asked about the activities that continued non-stop for a minimum of 10 min. The responses made it possible to compute the Metabolic Equivalent of Tasks minutes per week (MET-min/week) to gain an overall estimate of physical activity. One MET is defined as the energy expended while sitting quietly at rest, and is equivalent to 3.5 mL/kg/min of VO2 Max; it is used to assess specific types of activity. Each type of physical activity were converted into the units of MET-min/week by multiplying the coefficient assigned to that specific activity by the number of days it was performed per week, and the duration in minutes per day. Weekly physical activity was calculated based on the total results for each type of activity [15].

#### 2.3.2. Generalised Anxiety Disorder 7-Item (GAD-7 Scale)

Assessment of generalised anxiety disorder (GAD) in the respondents was based on the Generalised Anxiety Disorder 7-item (GAD-7) self-report questionnaire designed to screen for anxiety in primary care. It is one of the most frequently used tools for GAD screening worldwide. It is widely recognised as it is based on the associated symptom criteria listed in the Diagnostic and Statistical Manual of Mental Disorders, 4th ed., (DSM-IV) [16]. The tool comprises questions related to seven mental states, i.e., feeling nervous, anxious, or on edge; being able to stop or control worrying; worrying too much about different things; trouble relaxing; being restless; becoming easily annoyed or irritable; and feeling afraid as if something awful might happen. The responses are based on a 4-point Likert scale; more specifically each item is rated by the respondent on a scale from 0 to 3, with a possible maximum total score of 21 points. Higher scores reflect more severe anxiety. The cut-off scores for minimal, mild, moderate, and severe anxiety symptoms are 0–4, 5–9, 10–14, and 15–21, respectively. A score of 10 points corresponds to a significant likelihood of GAD [17]. 

#### 2.3.3. Specially Designed Questionnaire

The initial part of the online questionnaire addressed socio-demographic and socioeconomic characteristics (such as age, sex, education, place of residence), this way it was possible to perform a reliable selection to ensure that a homogenous group of young adults was taken into account in the analyses.

### 2.4. Statistical Analysis Methods

Statistical analyses of the collected material were computed in Statistica 13.1 package (StatSoft Polska, Cracow, Poland). Analyses of the variables were conducted using non-parametric tests exclusively. The choice of this type of tests was determined by the failure to meet the basic assumptions of parametric tests, i.e., the lack of agreement between the distributions of the variables and normal distribution, assessed using a Shapiro-Wilk W test. Descriptive statistics, computed for majority of the variables included: mean, median, minimum, maximum, First, and Third quartile, as well as standard deviation. Assessment of intragroup variability was performed using Wilcoxon signed-rank test. Correlations between two variables failing to meet the criterion of normal distribution were determined using Spearman’s rank correlation coefficient. Pearson’s chi-squared test was applied to assess the relationships between variables of qualitative nature. Significance was assumed at *p <* 0.05.

## 3. Results

### 3.1. Characteristics of the Study Group

Mean age of the respondents was 24.67 years ± 4.23 years (18–34 years). Responses to the survey were more frequently submitted by women, accounting for 74.9% of the study group, and normative body weight was reported by the largest group, i.e., 71.3% of respondents. More than one in two respondents, i.e., 54.5%, were residents of urban areas and a majority of 52% reported secondary formal education. Table 1 presents the study group characteristics (Table 1).

### 3.2. Changes in the Level of Physical Activity

Comparative analyses were performed to assess respondents’ PA before the COVID-19 pandemic and at the time of the lockdown during the pandemic. The results in each separate category and the overall findings differed significantly in the two periods of time (*p <* 0.001). During the pandemic, the respondents expended less energy performing vigorous and moderate PA, they walked far less frequently and reported lower overall PA. They spent significantly more time in a sitting position. Changes in all the investigated domains were significant (*p <* 0.001) (Table 2).

### 3.3. Changes in the Sense of Generalised Anxiety

There was a difference in the level of generalised anxiety experienced by the respondents in the period preceding the pandemic and during the pandemic. The analysis of the results showed that the level of generalised anxiety was significantly higher during the pandemic (*p <* 0.001) (Table 3).

### 3.4. Relationship between Physical Activity and Generalised Anxiety

There was a significant correlation between the level of generalised anxiety and physical activity reported by the respondents (*p =* 0.048) during the pandemic. A higher level of PA corresponded to a lower level of generalised anxiety. Conversely, the level of generalised anxiety was not significantly correlated to the level of PA in the respondents (*p =* 0.543) before the pandemic (Table 4).

More frequent walking and greater overall PA corresponded to a lower level of generalised anxiety in the respondents (*p =* 0.008, *p =* 0.043). Furthermore, before the outbreak of the pandemic, a greater amount of sedentary time corresponded to a higher level of generalised anxiety (*p =* 0.007) (Table 5).

## 4. Discussion

Mental disorders have become an increasingly serious health problem, with the dramatically growing incidence during 2000–2018, particularly in young adults. In 2019, anxiety and depression symptoms in the latter population were found at a rate of 20% and 21%, respectively [18,19]. Outbreak of the COVID-19 pandemic created unfavourable conditions, further exacerbating mental health issues, which is reflected in particular by the high rates of depressive disorder and anxiety disorders in young adults and in women [20]. Cumulative analyses from Europe, North America, Australasia, and Asia-Pacific showed that in 2020, following the outbreak of the pandemic, an additional 9.05 million cases of anxiety disorders were noted globally (an increase of 25.6%). Before the pandemic, prevalence of anxiety disorder amounted to 3824.9 cases per 100,000 population, which is equivalent to 298 million, whereas after the outbreak of the pandemic the numbers increased to 4802.4 cases per 100,000 population, equivalent to 374 million [21,22]. 

Regular physical activity (PA), sufficient for one’s age, has been identified as a potential therapeutic tool to reduce the risk of mental health problems in young adults [23]. So far, there is no consensus about the optimal amount of PA to relieve symptoms associated with mood and psychological disorders such as depression or anxiety. Some of the studies confirm that the most beneficial for mental health is moderate-intensity PA [24], and it is enough to make a positive difference in feeling good mood and well-being. It is established that neurobiological effects of exercise influence an increase in levels of chemicals in the brain (such as serotonin, stress hormones, endorphins, and neurotrophic factors) whose role is important in reduction of depressive and anxiety disorders. The higher-intensity exercise produces larger increases in peripheral neurotrophin levels [25,26].

The current findings confirm that, also in Poland, the outbreak of the pandemic resulted in decreased physical activity and increased generalised anxiety in young adults, believed to be a potentially healthy population, and statistical analyses confirmed the correlation between the two domains. Significant decline in physical activity during the first pandemic lockdown, when people were forced to adopt more sedentary lifestyles and spend most of their time at home, was also reported by many researchers, e.g., Luciano [27], Thomas [28], Baceviciene [29], and Tornaghi [30]. The differences between the two periods investigated in the present study were highly significant in all the measures, i.e., vigorous and moderate physical activity, walking, and total physical activity (*p <* 0.001). The mean sedentary time increased from 4 to 5 h daily. As a comparison, Romero-Blanco et al. reported that Spanish students during lockdown increased they daily sitting time by approximately 2.5 h [31]. A study by Karageorghis et al. presented interesting comparative data related to the populations of the USA, UK, France, and Australia during the first wave of international lockdowns. Across the four nations, daily step count decreased by 2000, whereas the reported sitting time increased by 2 h. Young adults were far less likely to engage in unplanned PA during lockdown, compared to other age groups, with the most marked decline evident in Europe [32]. Likewise, in a Chinese population, Qin reported the highest prevalence of insufficient PA in young adults aged 20–34 years, compared to other age groups [33].

The current study showed that a decrease in PA corresponded to greater sense of distress and anxiety symptoms of varied severity, predisposing for GAD. According to the Diagnostic and Statistical Manual of Mental Disorders, 4th Edition, Text Revision (DSM-IV-TR), individuals with GAD tend to experience excessive anxiety and constantly worry for at least six months. The anxiety is exaggerated and does not match the actual threat. Frequent symptoms associated with anxiety include problems with attention, high susceptibility to fatigue, inability to sit quietly, tension headaches, muscle pain, irritability, increased sweating, and a high heart rate [34]. The present findings show the highest increase in the number of individuals reporting a severe (from 1.7% to 7%), moderate-to-severe (from 4.9% to 11.5%), and a moderate level of generalised anxiety (from 18.8% to 32.1%); these changes were significant. Other reports from Poland related to the same period suggest the same trend. Babicki reported varied intensity of anxiety symptoms in nearly three in four subjects (71%), with as many as 23% of the study participants exhibiting a severe form of anxiety disorder, and 44% presenting characteristics of GAD. According to other Polish researchers, epidemiological studies from the times before the pandemic reported that signs of GAD, more frequently observed in women, overall were found in only 1% of the Polish population, which reflects the fact that the pandemic seriously exacerbated this problem [35]. Gambin et al. emphasised that the most severe symptoms of depression and anxiety were identified in subjects < 45 years of age, particularly in young adults aged 18–24 years. Other authors also observed that anxiety, depression, stress, and GAD were highly prevalent especially among young adults (≤24 years), with no corresponding increase in treatment seeking [36,37]. During the peak of the first pandemic wave, in Turkey as many as 44.5% young adults, according to the GAD-7 scale, had moderate/severe anxiety symptoms [38], whereas in Nigeria 34% and 9.3% of the subjects had moderate and severe anxiety symptoms, respectively [39]. Similarly, a high rate of 44.1% was identified in Brazil [40]. Evidence from the UK suggests that the number of young people experiencing anxiety had almost doubled, with a rate of 24% compared to a pre-pandemic level of 13% [41]. During the pandemic, GAD was also identified in young adults at a rate of 20.7% in Iran [42], and 10.9% in the USA [43]. Conclusions presented by many researchers are similar. It can be assumed that young people suffered the most negative impact from the viewpoint of their mental and physical functioning because their specific needs, such as autonomy, meeting with friends, travelling, and education were most seriously affected by the restrictions and the necessity to switch to remote communication, and distance learning or work. Perhaps the lifestyles of people aged >45 years to a lesser degree involve going out (to restaurant, club, gym, or university). Middle-aged or older adults also have more stable occupational and family status. Furthermore, being a parent of a child under 18 years of age was an important predictor of depression symptoms at a later stage of the epidemic [44]. 

The phenomenon of the significantly decreased level of PA during the pandemic made it possible to investigate the association between PA and mental wellbeing, including the incidence of GAD in young people. It has generally been established that regular PA results in many physiological changes and adaptations in the human body which prevent many stress-related disorders, such as anxiety, depression, agoraphobia, post-traumatic stress disorder, and panic disorder [45]. Improvement of mood induced by PA results from increased secretion of such neurotransmitters as endorphins and monoamines, which reduce pain and trigger positive feelings [46]. Regular aerobic exercise beneficially affects the reactivity of the sympathetic nervous system and the hypothalamic-pituitary-adrenal (HPA) axis. The HPA axis is responsible for adaptive responses to physical and psychological stressors, whereas any deregulation of its functions may contribute to the development of anxiety- and depression-related disorders [47]. It has also been demonstrated that PA beneficially affects endogenous opioid activity in the central and peripheral nervous system, which may reduce the feeling of pain. This may be an important finding for preventing mental diseases since endogenous opioids have a role in the regulation of mood and emotional responses [48]. It has been demonstrated that individuals with high anxiety sensitivity to physiological symptoms increase their tolerance to these types of sensations as a result of regular exercise [49]. Although there is strong evidence that PA improves mental well-being, and potentially may prevent symptoms of mental health disorders, most related research involves populations of adults and elderly people, whereas the evidence of any such relationships in younger cohorts is not consistent [50]. 

The present findings show that a higher level of PA corresponded to a less pronounced sense of generalised anxiety in young adults. This relationship was only observed in the responses related to the situation during the lockdown. Perhaps more up-to-date research carried out after the outbreak pandemic will provide stronger evidence for the relationship between PA and mental health disorders. Wolf et al. performed a systematic review taking into account 21 observational studies related to this issue, and they estimated that a risk of depressive symptoms was 12–32% lower and a risk of anxiety disorder was 15–34% lower in the subjects reporting a higher total time spent in moderate to vigorous PA [51]. Marconcin et al. reviewed studies related to the first lockdown and showed that most findings suggest that sufficiently active participants reported significantly lower depression and anxiety and a higher life satisfaction. There is mixed evidence regards to the most effective amount of PA, however it has been assumed that more vigorous and frequent PA corresponds to a better mental condition [52]. Many research reports which have been published focus particularly on young adults, like the current study. According to Faulkner et al., the negative change in exercise behaviour, commonly observed in individuals aged 18–29 years, compared to pre-COVID-19 period with no restrictions, was associated with poorer mental health and well-being [53]. Stanton et al. also pointed out that the highest risk of one or more psychological distress states was observed in the case of individuals ranging in age from 18 to 45 years and insufficient physical activity, lack of sleep as well as smoking and alcohol intake were associated with higher depression, anxiety, and stress symptoms [54].

Preventing mental diseases, such as GAD, in the stressful contemporary world (particularly during the pandemic), is a challenging task which must be handled by public health specialists. Measures which should be taken to prevent GAD in young adults, besides pharmacotherapy and psychotherapy, must focus on the promotion of active lifestyles, and sufficient levels of PA. In contemporary times, measures aimed to prevent mental diseases should also address young adults, who generally are not seen as a potential risk group. The time of COVID-19 pandemic highlights the related vulnerabilities making it necessary to initiate additional action [55].

The presented study has some limitations. The presented data focused on the positive role of PA, but inactivity can be both a cause and a consequence of mental illness. The study did not explore the role of associated factors like gender, BMI, place of residence, or level of physical activity. There was an inability to use objective research methods, requiring direct contact with the subject during the spread of COVID-19 pandemic. It would be also advisable to find the minimum and optimal PA levels for the improvement of mental health in young people.

## 5. Conclusions

Physical activity contributes to reducing anxiety disorder in young adults. During the COVID-19 pandemic young adults were significantly less involved in PA, which adversely affected their physical and mental condition. The importance of sufficient PA should be highlighted during the specific period of the COVID-19 pandemic, particularly among young adults.

## Figures and Tables

**Table 1:** Characteristics of the study group.

Characteristics of the Study Group		N	%
**Age**	24.67 ± 4.23 years (18–34 years)		
**Sex**	Female	379	74.9%
	Male	127	25.1%
**BMI Category**	Underweight	26	5.1%
	Normal	361	71.3%
	Overweight	90	17.8%
	Obese	29	5.7%
**Place of residence**	Countryside	230	45.5%
	Town < 10 thousand residents	56	11.1%
	Town 10–100 thousand residents	96	19.0%
	City 100–300 thousand residents	95	18.8%
	City 300 thousand-1 million residents	29	5.7%
**Education**	Secondary	263	52.0%
	Basic vocational	10	2.0%
	Higher	233	46.0%

N—number of participants; %—percent.

**Table 2:** The level of physical activity before and during the pandemic.

IPAQ [MET-Min/Week]	Period of 7 Days during First Hard Lockdown			Period of 7 Days before Pandemic			Z	*p*
	Me	Q1	Q3	Me	Q1	Q3		
**Intensive PA**	0.0	0.0	960.0	480.0	0.0	1920.0	7.31	<0.001
**Moderate PA**	240.0	0.0	720.0	360.0	0.0	840.0	4.10	<0.001
**Walking**	3861.0	1485.0	7260.0	6930.0	3762.0	9702.0	11.35	<0.001
**Total PA**	5483.0	2380.0	9009.0	8752.5	5403.0	11,820.0	12.39	<0.001
**Sedentary time [min/day]**	300.0	180.0	420.0	240.0	120.0	360.0	6.99	<0.001

Z—Wilcoxon matched-pairs signed rank test result; *p*—probability level test.

**Table 3:** The level of generalised anxiety before and during the pandemic.

GAD-7 [pkt.]	Descriptive Statistics							
	**N**	$\bar{x}$	**Me**	**Min.**	**Max.**	**Q1**	**Q3**	**SD**
**Period of 7 days during first hard lockdown**	506	6.43	6.00	0.00	23.00	2.00	9.00	5.40
**Period of 7 days before pandemic**	506	3.68	3.00	0.00	21.00	0.00	6.00	4.07
**Significance (*p*)**	Z = 11.33 *p <* 0.001							

N—number of participants; $\bar{x}$ —average, Me—median value; Min.—minimum; Max.—maximum; Q1—Quartile 1; Q3—Quartile 3; SD—standard deviation; *p*—probability level test.

**Table 4:** Correlations between the level of generalised anxiety and physical activity before and during pandemic.

	Period of 7 Days during First Hard Lockdown							
**The Level of Generalised Anxiety**	**High Level of PA**		**Sufficient Level of PA**		**Insufficient Level of PA**		**Total**	
	**N**	**%**	**N**	**%**	**N**	**%**	**N**	**%**
**Mild**	206	49.4%	18	51.4%	18	34.0%	242	47.9%
**Moderate**	134	32.1%	12	34.3%	20	37.7%	166	32.9%
**Moderately severe**	48	11.5%	4	11.4%	5	9.4%	57	11.3%
**Severe**	29	7.0%	1	2.9%	10	18.9%	40	7.9%
**Total**	417	100.0%	35	100.0%	53	100.0%	505	100.0%
**Significance (*p*)**	χ^2^(6) = 12.74 *p =* 0.048							
	**Period of 7 Days before Pandemic**							
**Mild**	350	74.6%	10	66.7%	17	77.3%	377	74.5%
**Moderate**	88	18.8%	3	20.0%	2	9.1%	93	18.4%
**Moderately severe**	23	4.9%	2	13.3%	2	9.1%	27	5.3%
**Severe**	8	1.7%	0	0.0%	1	4.5%	9	1.8%
**Total**	469	100.0%	15	100.0%	22	100.0%	506	100.0%
**Significance (*p*)**	χ^2^(6) = 5.01 *p =* 0.543							

PA—physical activity; N—number; %-percent; χ^2^—Pearson’s chi-square test result; *p*—test probability value.

**Table 5:** Associactions between GAD-7 total score and physical activity during and before pandemic.

Associations during First Hard Lockdown (Period of 7 Days)	R	*p*
GAD-7 total score vs. Intensive PA	0.00	0.922
GAD-7 total score vs. Moderate PA	0.05	0.267
GAD-7 total score vs. Walking	−0.12	0.008
GAD-7 total score vs. Sedentary time	0.09	0.051
GAD-7 total score vs. Total PA	−0.09	0.043
**Associations Before Pandemic (Period of 7 Days)**	**R**	***p***
GAD-7 total score vs. Intensive PA	−0.01	0.873
GAD-7 total score vs. Moderate PA	0.04	0.414
GAD-7 total score vs. Walking	−0.07	0.109
GAD-7 total score vs. Sedentary time	0.12	0.007
GAD-7 total score vs. Total PA	−0.06	0.152

PA—physical activity; R—Spearman rank correlation; *p*—probability level test.

## Data Availability

Data available from the authors of this publication.
